# *Wolbachia* diversity and cytoplasmic incompatibility patterns in *Culex pipiens* populations in Turkey

**DOI:** 10.1186/s13071-018-2777-9

**Published:** 2018-03-20

**Authors:** Mine Altinli, Filiz Gunay, Bulent Alten, Mylene Weill, Mathieu Sicard

**Affiliations:** 1 Institut des Sciences de l’Evolution de Montpellier (CNRS-Université de Montpellier-IRD-EPHE), Montpellier, France; 2 Faculty of Sciences, Department of Biology, Division of Ecology, VERG Laboratories, Hacettepe University, Ankara, Turkey

**Keywords:** *Wolbachia*, *Culex pipiens*, Cytoplasmic incompatibility, Turkey, Vector control

## Abstract

**Background:**

*Wolbachia* are maternally transmitted bacteria that can manipulate their hosts’ reproduction causing cytoplasmic incompatibility (CI). CI is a sperm-egg incompatibility resulting in embryonic death. Due to this sterilising effect on mosquitoes, *Wolbachia* are considered for vector control strategies. Important vectors for arboviruses, filarial nematodes and avian malaria, mosquitoes of *Culex pipiens* complex are suitable for *Wolbachia-*based vector control. They are infected with *Wolbachia w*Pip strains belonging to five genetically distinct groups (*w*Pip-I to V) within the *Wolbachia* B supergroup. CI properties of *w*Pip strongly correlate with this genetic diversity: mosquitoes infected with *w*Pip strains from a different *w*Pip group are more likely to be incompatible with each other. Turkey is a critical spot for vector-borne diseases due to its unique geographical position as a natural bridge between Asia, Europe and Africa. However, general *w*Pip diversity, distribution and CI patterns in natural *Cx. pipiens* (*s*.*l*.) populations in the region are unknown. In this study, we first identified *w*Pip diversity in Turkish *Cx. pipiens* (*s*.*l*.) populations*,* by assigning them to one of the five groups within *w*Pip (*w*Pip-Ito V). We further investigated CI properties between different *w*Pip strains from this region.

**Results:**

We showed a *w*Pip fixation in *Cx. pipiens* (*s*.*l*.) populations in Turkey by analysing 753 samples from 59 sampling sites. Three *w*Pip groups were detected in the region: *w*Pip-I*, w*Pip-II and *w*Pip-IV. The most dominant group was *w*Pip-II. While *w*Pip-IV was restricted to only two locations, *w*Pip-I and *w*Pip-II had wider distributions. Individuals infected with *w*Pip-II were found co-existing with individuals infected with *w*Pip-I or *w*Pip-IV in some sampling sites. Two mosquito isofemale lines harbouring either a *w*Pip-I or a *w*Pip-II strain were established from a population in northwestern Turkey. Reciprocal crosses between these lines showed that they were fully compatible with each other but bidirectionally incompatible with *w*Pip-IV Istanbul infected line.

**Conclusion:**

Our findings reveal a high diversity of *w*Pip and CI properties in *Cx. pipiens* (*s*.*l*.) populations in Turkey. Knowledge on naturally occurring CI patterns caused by *w*Pip diversity in Turkey might be useful for *Cx. pipiens* (*s*.*l*.) control in the region.

## Background

First discovered in *Culex pipiens* (*s*.*l*.) mosquitoes [1], the α-proteobacterium *Wolbachia pipientis* is one of the most common vertically transmitted cytoplasmic symbionts. Indeed, meta-analysis predicts *Wolbachia* infection in up to 50% of the arthropod species [2]. The success of their vertical transmission mainly relies on their host reproduction manipulation strategies; parthenogenesis induction, feminization, male killing and cytoplasmic incompatibility (CI) [3]. CI, the most common of these strategies, is modelled by a modification-rescue (*mod*-*resc*) system where *Wolbachia* modifies sperm of infected males (*mod* function), and only a compatible *Wolbachia* strain in the eggs can rescue (*resc* function) this modification [4]. Consequently, *Wolbachia* causes conditional sterility in crosses either between uninfected females and infected males [5] or like in the case of *Cx. pipiens* (*s*.*l*.), between females and males infected with incompatible *Wolbachia* strains [6, 7].

Consisting of several species, including important disease vectors with worldwide distribution (e.g. *Cx. pipiens* and *Cx. quinquefasciatus*) [8], mosquitoes of *Cx. pipiens* complex have a unique relationship with their endosymbiont *Wolbachia* (*w*Pip). *w*Pip is fixed in natural *Cx. pipiens* (*s*.*l*.) populations where they induce the most complex CI relationships yet described among arthropods, including uni and bidirectional incompatibility [7, 9–12]. So far, reciprocal crosses between many isofemale lines and four reference lines showed eight different *mod* and four different *resc* functions in *Cx. pipiens* (*s*.*l*.), resulting in the definition of 14 different cytotypes throughout the world [7].

In contrast to this observed diversity of CI patterns, *Cx. pipiens w*Pip strains are closely related, and all belong to a clade within *Wolbachia* B supergroup [12–14]. However, recent studies of fast evolving markers showed the presence of many genetically distinct *w*Pip strains in *Cx. pipiens* (*s*.*l*.) mosquitoes [10, 12, 15] distributed in five distinct phylogenetic groups (*w*Pip-I to V) [12]. Using a PCR/RFLP assay based on pk1 gene, encoding proteins with ankyrin motifs, a *w*Pip strain can be assigned to one of these five groups [12, 16]. A study of the *w*Pip worldwide distribution showed an important spatial structure of *w*Pip groups [16]. For instance, only *w*Pip-I was found in sub-Saharan Africa, South America and Southeast Asia, while *w*Pip-III was mainly observed in North America. Strains belonging to the *w*Pip-II group were mostly found in western Europe and *w*Pip-V in Asia. *w*Pip-IV group strains exhibit a patchy distribution in Europe, North Africa and Asia [16]. Also, *Wolbachia* genetic diversity and their CI patterns strongly correlate; most *w*Pip strains from the same group render their host compatible with each other (except few unidirectional incompatibilities) whereas those from different groups often lead to unidirectional or bidirectional incompatibilities [7]. Recently this huge diversity of CI patterns observed in *Cx. pipiens* has been explained by the amplification and the diversity of an operon in *w*Pip strains’ genomes [17] composed of *cidA* and *cidB* genes involved in *Wolbachia* induced CI [18, 19]. No effect of host genetic background on the CI patterns [20] and no multiple infections by several strains have ever been shown [12, 16, 21].

Being a natural bridge between Africa, Asia and Europe, Turkey is a critical spot for many emerging and re-emerging vector-borne diseases [22, 23] and for the diversity of the vectors that transmit these diseases [23]. For instance, high diversity and abundance of *Cx. pipiens* (*s*.*l*.) species have been recorded in the area including *Cx. quinquefasciatus*, *Cx. pipiens* and its physiological variant *Cx. pipiens* f. *molestus* [24]. Arboviruses such as West Nile virus, mainly transmitted by these mosquitoes, have also been shown to circulate in Turkey [25–28]. Therefore, understanding *w*Pip diversity and their CI properties of *w*Pip to control *Cx. pipiens* (*s*.*l*.) populations in Turkey is a cornerstone for vector control in the region and prevention of putative epidemics extending through Europe, Asia and northern Africa. This knowledge can contribute to the biological vector control techniques using CI properties such as incompatible insect technique (IIT). IIT, the mass release of males harbouring incompatible *Wolbachia* into focal populations, has been shown to successfully decrease the female reproduction by sterilisation and reduce the pest/vector populations [29–34]. Nevertheless, our knowledge of the *w*Pip genetic diversity and the CI patterns in Turkey is yet limited to only one line established with samples collected in Istanbul in 2003 [35].

Here, we collected and analysed 753 *Cx. pipiens* (*s*.*l*.) individuals (larvae and adults) from natural populations across Turkey. We studied (i) the *w*Pip diversity in this geographically critical region in the crossroads of three continents, (ii) the CI relationships between *Cx. pipiens* lines from Turkey and (iii) the CI relationships between Turkish lines and reference lines to compare their CI properties to previously characterized *mod* (male crossing type) and *resc* (female crossing type) functions. Taken together, these results might be used in integrated vector control programs against *Cx. pipiens* (*s*.*l*.) in Turkey.

## Methods

### Sample collection and identification

**Fig. 1:**
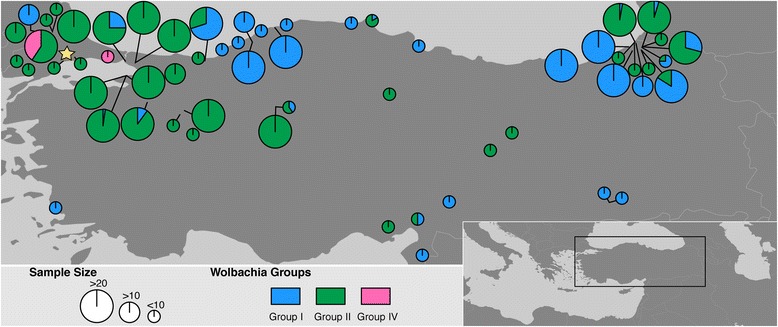
Sampling sites and diversity of *w*Pip in *Cx. pipiens* (*s*.*l*.) populations in Turkey. A total of 753 samples were collected from 59 different sampling sites, tested with a PCR/RFLP assay on the pk1 gene (1.3 kb) and assigned to one of the five genetically distinct *w*Pip groups (*w*Pip-I to V). Results showed the occurrence of *w*Pip from three different groups in the area namely *w*Pip-I-II and IV. Size of the circle represents the sampling size. Percentage of a given *w*Pip group in a given population is shown in different colours; blue: *w*Pip-I, green: *w*Pip-II, pink: *w*Pip-IV as defined in Dumas et al. [16]. Star indicates the location of the samples (Tekirdag, sampling site 52) that have been used to establish Tek *w*Pip-I and Tek *w*Pip-II lines. Reciprocal crosses between these lines and additional reference lines were performed to identify natural CI patterns caused by *w*Pip groups in this region

A total of 753 samples from 59 different sampling sites in Turkey were tested for *Wolbachia* diversity. Most of these samples (*n* = 677) were collected during the larval stage, between July to September 2016 (Table 1). The rest of the samples has been collected as adults, using adult light traps, from May to September (2012–2015) (Table 1, Fig. 1). All of the sampling sites were situated outdoors with the only exception of sample site 16 (Table 1). Collected larvae and adults were morphologically identified as *Culex pipiens* (*s*.*l*.) / *Cx. torrentium* [36]. As the samples were only morphologically identified, we used *Cx. pipiens* (*s*.*l*.) to refer to *Cx pipiens* assemblage that includes both *Cx. pipiens* and *Cx. quinquefasciatus*, their hybrids and physiological forms [21, 35]. Samples were stored in 70% ethanol until DNA extraction before testing them for the *w*Pip presence and genetic characterisation.

**Table 1:** Sampling sites, year, life stage and *w*Pip groups of *Culex pipiens* (*s.l.*) individuals collected from Turkey

Province	Sampling site	Latitude (°N)	Longitude (°E)	Breeding site type	Stage	Year	*w*Pip-I	*w*Pip-II	*w*Pip-IV
Adana	1	36.9475	35.485	Rural	A	2013	–	5	–
Ankara	2	39.8730	32.7370	Suburban	L	2016	–	27	–
	3	39.8716	32.7356	Suburban	A	2014	2	3	–
Artvin	4	41.3884	41.4335	Suburban	L	2016	1	35	–
	5	41.4919	41.5367	Suburban	L	2016	–	5	–
	6	41.3833	41.5716	Rural	L	2016	–	5	–
	7	41.3651	41.6835	Suburban	L	2016	1	20	–
	8	41.3911	41.6933	Rural	L	2016	7	17	–
	9	41.3646	41.6686	Suburban	L	2016	–	3	–
	10	41.3742	41.6235	Rural	L	2016	21	4	–
	11	41.3192	41.3534	Rural	L	2016	12	–	–
	12	41.3178	41.3412	Rural	L	2016	10	–	–
	13	41.3274	41.3022	Rural	L	2016	36	–	–
	14	40.7823	41.4991	Rural	A	2013	–	1	–
	15	41.3928	41.6937	Rural	A	2013	4	1	–
Aydin	16	37.4123	27.3612	Rural	A	2012	3	–	–
Bartin	17	41.8383	32.7115	Rural	L	2016	10	–	–
	18	41.7411	32.3827	Suburban	L	2016	5	–	–
Bursa	19	40.0948	29.4912	Urban	A	2013	1	9	–
Duzce	20	41.0708	30.9645	Rural	L	2016	7	3	–
Edirne	21	41.6134	26.9656	Rural	L	2016	–	16	11
	22	41.6731	26.9809	Rural	L	2016	11	–	–
	23	41.6635	26.5078	Suburban	L	2016	–	17	–
	24	40.8548	26.6897	Suburban	A	2012	–	4	–
	25	40.9404	26.4382	Rural	A	2012	–	1	–
Erzincan	26	39.2476	38.5050	Rural	A	2014	–	3	–
Eskisehir	27	39.7950	30.4972	Urban	L	2016	–	27	–
	28	39.2051	30.7145	Rural	A	2013	–	2	–
	29	39.7098	30.4035	Rural	L	2016	–	5	–
Hatay	30	36.2516	36.3166	Suburban	A	2015	1	–	–
Istanbul	31	40.9481	29.3050	Urban	L	2016	–	38	–
	32	40.9418	29.3016	Urban	L	2016	–	44	–
	33	40.9796	29.0557	Urban	L	2016	8	24	–
	34	41.0783	29.0136	Urban	A	2016	–	–	1
Kahramanmaras	35	37.5588	36.9737	Urban	A	2015	3	–	–
Karadeniz Ereglisi	36	41.2824	31.4241	Urban	L	2016	6	–	–
Kastamonu	37	41.8886	32.9995	Suburban	L	2016	5	–	–
Kirklareli	38	41.8458	27.8065	Rural	L	2016	–	10	–
	39	41.5239	27.0258	Rural	A	2015	–	6	–
	40	41.8300	27.0638	Rural	A	2015	–	2	–
Kocaeli	41	40.6882	30.2797	Urban	L	2016	–	15	–
Malatya	42	38.8180	37.9769	Rural	A	2014	–	1	–
Mardin	43	37.5607	40.8865	Rural	A	2013	5	–	–
	44	37.5477	40.9588	Rural	A	2013	5	–	–
Osmaniye	45	37.1375	36.2010	Suburban	A	2015	1	1	–
Sakarya	46	41.0719	30.8454	Suburban	L	2016	–	5	–
Samsun	47	41.3689	36.2289	Urban	A	2013	3	–	–
Sinop	48	41.9915	35.0908	Suburban	L	2016	1	5	–
	49	41.9309	34.5819	Suburban	L	2016	6	–	–
Tekirdag	50	41.1503	27.8522	Urban	A	2012	–	3	–
	51	40.8886	27.4604	Urban	A	2015	–	6	–
	52^a^	41.0259	27.5805	Urban	L	2016	na	na	na
Tokat	53	40.1916	35.5139	Rural	A	2014	–	1	–
Trabzon	54	40.8938	39.7113	Suburban	L	2016	34	–	–
Yalova	55	40.6217	29.1770	Rural	L	2016	1	44	–
	56	40.6085	29.2080	Suburban	L	2016	–	42	–
	57	40.6428	29.0968	Suburban	L	2016	–	40	–
Zonguldak	58	41.4105	32.0890	Suburban	L	2016	10	–	–
	59	41.3537	32.0900	Suburban	L	2016	15	–	–
	60	41.4529	31.8203	Urban	L	2016	7	–	–

*Abbreviations*: A, adult; L, larva; na, not applicable

*Note*: Columns *w*Pip-I, *w*Pip -II and *w*Pip-IV indicate the amount of individuals infected with *w*Pip-I, *w*Pip -II and *w*Pip-IV, respectively, in a given sampling site

^a^Samples from this site were used to establish Tek *w*Pip-I and Tek *w*Pip-II lines

### Isofemale lines

For analysing the CI patterns induced by the *w*Pip strains belonging to different groups found in Turkey, egg rafts and larvae were collected from a population in Thrace region of Turkey, in Tekirdag (Table 1 sampling site 52, Fig. 1). Collected larvae were reared to adults in insectary conditions (at 25 ± 2 °C and 75 ± 2% relative humidity and a 12:12 h photoperiod) and fed with a mixture of shrimp powder and rabbit pellets. To establish isofemale lines, females were fed with turkey blood using a Hemotek membrane feeding system (Discovery Workshops, Blackburn, United Kingdom) and were allowed to lay eggs five days later. Each egg raft (100–300 eggs) was isolated for hatching, and the isofemale line was established using resulting sibling larvae. A pool of first-instar larvae (L1) was tested to identify the *w*Pip group. Two isofemale lines each harbouring either a *w*Pip-I or *w*Pip-II strain were reared for further crossing experiments in 65 dm^3^ cages in insectary conditions and were fed with a honey solution and a weekly blood meal.

### Crossing experiments between Turkish *Culex pipiens* lines

Tek *w*Pip-I and Tek *w*Pip-II isofemale mosquito lines were reared for at least four generations in insectary conditions to allow their acclimatisation before crossing experiments. Mosquitoes were isolated during pupal stage, and emerging adults were sexed. Then, 2–5 days old virgin males and females (*n* = 25–50) were used to carry out reciprocal crosses between them and with Istanbul *w*Pip-IV line. Females were fed with turkey blood using a Hemotek membrane feeding system (Discovery Workshops, United Kingdom) on the sixth day following caging and were allowed to lay eggs five days after the blood meal. Egg rafts were then isolated individually in 24 well plates filled with tap water until hatching. Embryonic development of all the unhatched egg rafts was verified to differentiate between non-fertilized egg rafts and CI induced embryonic death as previously described [37].

The crossing relationships were identified as following [7]: compatible (C) when > 90% of the rafts hatched in the two reciprocal crosses; and incompatible (IC), with two CI patterns: (i) unidirectionally incompatible crosses: when between 0–10% of the rafts hatched in one of the reciprocal crosses and > 90% in the other; and (ii) bidirectionally incompatible crosses: when less than 10% of the rafts hatched in both reciprocal crosses.

### Crossing experiments to infer *mod* and *resc* functions

The *mod* (male crossing type) and *resc* (female crossing type) functions caused by many *w*Pip strains, which belong to different *w*Pip groups (I-V), have been identified by reciprocal crosses with 4 reference lines: LaVar (*w*Pip-II), MaClo, Slab (*w*Pip-III) and Istanbul (*w*Pip-IV) [7]. Here, we used same four reference lines to define the *mod-resc* functions of Tek *w*Pip-I and Tek *w*Pip-II isofemale lines and to compare them to previously defined ones [7].

### Identification of *Wolbachia* diversity

DNA was extracted from the samples using CTAB method [38]. PCR assays were conducted using pk1 primers (PK1 Forward: 5'-CCA CTA CAT TGC GCT ATA GA-3' and PK1 Reverse: 5'-ACA GTA GAA CTA CAC TCC TCC A-3'-AM397079 [12]), which amplify a 1.3-kilobase (kb) fragment from ankyrin domain coding gene of *Wolbachia*. PCR amplifications were made in following conditions: initial denaturation for 5 min at 94 °C, followed by 35 cycles of denaturation, annealing and elongation respectively at 94 °C for 30 s, 52 °C for 30s, and 72 °C for 90 s, and a final elongation at 72 °C for 5mn. Resulting PCR products then used in RFLP assays first with *Taq*αI enzyme to discriminate specific *w*Pip alleles “a” or “e” (*w*Pip- I or *w*Pip-V; 991*,* 251, 107 bp), “b” (*w*Pip-III; 669, 665 bp), “c” (*w*Pip-II; 851, 498 bp) and “d” (*w*Pip-IV; 497, 251, 107 bp) [7, 16]. Secondly, since *Taq*αI digestion of “a” and “e” alleles show the same digestion pattern, pk1 PCR products of the samples showing this pattern were digested with *Pst*I enzyme to further discriminate “a” (*w*Pip- I; 903, 303, 141 bp) and “e” (*w*Pip-V; 903, 430 bp) alleles [7, 16]. Digested amplified fragments were separated by agarose gel electrophoresis (2%), stained with ethidium bromide (1 μg/ml) and visualized with UV light. Samples from laboratory mosquito lines with different *w*Pip groups and tetracycline-cured *Wolbachia* negative lines were included in every reaction as positive and negative controls, respectively, and always gave the expected result.

### Statistical analyses

The occurrence of different *w*Pip groups was compared by a Chi-square test using R software (version 3.3.1).

## Results

### Diversity and distribution of *w*Pip groups

*Wolbachia w*Pip was present in all of the 753 *Cx. pipiens* (*s*.*l*.) individuals tested and they were further identifiable to one of the five previously described groups (*w*Pip-I to V). Co-infection of one individual by different *w*Pip groups was never observed. Out of five *w*Pip groups identified so far in the world, three of them (i.e. *w*Pip-I-II and IV) were represented in the studied area (Fig. 1). The abundance of these groups was significantly different from each other (*χ*^2^ = 474.99, *df* = 2, *P* < 0.0001). While *w*Pip-II was the most dominant (*n* = 500, 66% of the samples, Table 1, Fig. 1) and widespread group (39 sampling sites out of 59 total) in Turkey; *w*Pip-IV was found only in two locations, both in Thrace Region (in Edirne, sampling site 21 and Istanbul sampling site 34, Table 1; Fig. 1), and was the least abundant group (*n* = 12, 1% of the samples, Table 1; Fig. 1). *w*Pip-I was found in 31 locations and a total of 241 individuals.

### Co-existence of *w*Pip strains in different individuals from the same sampling sites

In 20 % of the sampling sites *w*Pip-I and *w*Pip-II co-existed (Table 1, Fig. 1). *w*Pip-IV was only found co-existing with *w*Pip-II in one sampling site but never found in the same sampling site with *w*Pip-I, even though they were sampled from nearby sites (~8 km) in north western Turkey (in Edirne, sampling site 21 and 23, Table 1; Fig. 1).

### Naturally occurring CI patterns in Turkey

Two isofemale lines (Tek *w*Pip-I and Tek *w*Pip-II), harbouring two different *w*Pip strains from two different groups, were established from north western Turkey (Tekirdag Province, Table 1 sampling site 52, Fig. 1) to identify CI patterns caused by different *w*Pip groups in the region. Reciprocal crosses between these lines showed that Tek *w*Pip-I and Tek *w*Pip-II were fully compatible with each other (Table 2). Both lines were bidirectionally incompatible with the line harbouring Istanbul strain (*w*Pip-IV, Table 2).

**Table 2:** Crossing relationships between lines from Turkey (Tek *w*Pip-I & Tek *w*Pip-II) and reference laboratory *Wolbachia* strains

	Males	Tek *w*Pip-I	Tek *w*Pip-II	Istanbul *w*Pip-IV	Slab *w*Pip-III	LaVar *w*Pip-II	MaClo *w*Pip-III
	*Mod*	vi	ix	viii			
Females	***Resc***						
Tek I	**2**		C (24)	**IC (24)**	C (9)	C (17)	C (34)
Tek II	**2**	C (26)		**IC (32)**	C (26)	C (20)	C (14)
Istanbul	**3**	**IC (58)**	**IC (36)**		IC (34)^a^	**IC (40)** ^a^	C (31)^a^
Slab		**IC (32)**	C (27)	C (33)^a^		IC (30)^a^	IC (99)^a^
LaVar		C (15)	IC (33)	**IC (26)** ^a^	C (8)^a^		C (10) ^a^
MaClo		C (18)	C (20)	IC (53)^a^	C (43)^a^	C(36)^a^	

^a^Data taken from Duron et al. [10]

*Note*: Reciprocal crosses between Tek *w*Pip-I, Tek *w*Pip-II, Istanbul *w*Pip-IV lines have been performed to identify natural CI patterns induced by these strains in the region. Additional reciprocal crosses between Turkish lines and 4 reference laboratory lines [LaVar (*w*Pip-II), MaClo and Slab (*w*Pip-III) and Istanbul (*w*Pip-IV)] have been performed to define the *mod-resc* functions of Tek *w*Pip-I and Tek *w*Pip-II isofemale lines and to compare them to previously defined ones by Atyame et al. [7]. Crosses were classified as either compatible (C, raft hatching > 90%) or incompatible (IC, raft hatching = 0–10%). Bidirectionally incompatible crosses are shown in bold. The number of egg-rafts collected for each cross is indicated in parentheses

### *Mod* and *resc* properties of Turkish *w*Pip strains

To compare *mod* and *resc* functions of Turkish *w*Pip strains with *mod* and *resc* functions of worldwide collected *w*Pip strains, we performed reciprocal crosses of Tek *w*Pip-I and Tek *w*Pip-II lines with the four reference lines [LaVar (*w*Pip-II), MaClo (*w*Pip-III), Slab (*w*Pip-III) and Istanbul (*w*Pip-IV)]. Tek *w*Pip-I males were compatible with LaVar (*w*Pip-II) and MaClo (*w*Pip-III) females while incompatible with Slab (*w*Pip-III) and Istanbul (*w*Pip-IV) females (Table 2). This type of *mod* property, inferred from similar crosses, has already been shown for the *w*Pip-I group from Tunisia; numbered *“*vi” [7]. Contrarily, Tek *w*Pip-II males demonstrated a new *mod* property, as they were incompatible with LaVar and Istanbul, and compatible with MaClo and Slab females (Table 2). We numbered this new *mod* as “ix” to continue the previously published numeration [7]. Both Tek *w*Pip-I and Tek *w*Pip-II lines showed the same *resc* type, which was characterised by the compatible crosses of females of these lines with all the males from the reference lines except Istanbul (Table 2). This *resc* type (*resc* “2”) is the most common *resc* type found worldwide for *w*Pip-I and *w*Pip-II groups [7].

## Discussion

In Turkey, all tested *Cx*. *pipiens* were infected with *Wolbachia w*Pip. Such fixation of *w*Pip has been demonstrated worldwide, including in the neighbouring country Iran [39], in *Cx. pipiens* and *Cx. quinquefasciatus* populations [35, 40–44]. A previous study in Turkey, however, showed a lower prevalence of *w*Pip [45]. This might be caused by the misidentification of a recently described cryptic species within *Cx. pipiens* complex that has been shown to lack *w*Pip infection and to be reproductively isolated from the other members of the complex [46, 47]. Similarly, *Cx. torrentium*, which is difficult to differentiate morphologically from *Cx. pipiens* (*s*.*l*.) mosquitoes is not infected with *Wolbachia* [41, 44, 48]. Therefore 100% *w*Pip infection rate of our samples confirmed that we only analysed *Cx. pipiens* complex members (excluding both previously mentioned cryptic species and *Cx. torrentium*) in the present study.

The identification of the *Cx. pipiens* taxa was left out of the scope of this study for several reasons. Previous studies on the diversification of *w*Pip in *Cx. pipiens* (*s*.*l*.) have proved that their diversity is not directly related to the nuclear genetic background of the mosquitoes, meaning that no *w*Pip group was specific for a *Cx. pipiens* sibling species [10, 21, 49]. It rather follows the same distribution as mitochondrial diversity (mtDNA) of mosquitoes, as *w*Pip are maternally transmitted to the next generation through the egg cytoplasm along with mitochondria [16, 46]. Moreover, CI properties are independent of the genetic background of *Cx. pipiens* (*s*.*l*.) and directly dictated by their *Wolbachia* [20]. Recent studies on *Cx. pipiens* (*s*.*l*.) in Turkey had shown that both *Cx. quinquefasciatus, Cx. pipiens* and its form *Cx. pipiens* f. *molestus*, were present in Turkey [24]. The co-existence of these sibling species in same sampling sites [24, 50] and the existence of hybrids [50–52] suggest that they can exchange *w*Pip strains easily in natural populations.

We have identified three different *w*Pip groups, i.e. *w*Pip-I-II and IV in *Cx. pipiens* (*s*.*l*.) mosquito populations in Turkey. The only previous sample from Turkey, which has been assigned to *w*Pip groups, was a *w*Pip-IV group strain collected in Istanbul in 2003 [16, 35]. Other than this single case, the *w*Pip diversity in Turkish *Cx. pipiens* populations was to date completely unknown. Although *w*Pip diversity was investigated in regions around Turkey [16]. Dumas et al. [16] have found *w*Pip-I strains in Middle East (Lebanon, Israel, Jordan) and in northern Africa (Tunisia), *w*Pip-II strains widely distributed in eastern Europe and Cyprus, and *w*Pip-IV strains in a patchy distribution in Europe -in areas dominated by other *w*Pip groups. We demonstrated that *w*Pip strains belonging to *w*Pip-I, *w*Pip-II and *w*Pip-IV, previously identified near Turkey, are all present in the *Cx. pipiens* (*s*.*l*.) populations within this country, suggesting that Turkey is a crossroads for *w*Pip strains from eastern Europe, Africa and Middle East as for their vector hosts.

The most widespread groups in Turkey, *w*Pip-I and *w*Pip-II, induce reciprocal compatibility between their hosts and co-exist in many populations. Indeed, different *w*Pip strains can co-exist in a single natural *Cx. pipiens* (*s*.*l*.) population [12, 35, 43, 53] and these coexisting strains are usually compatible with each other [43]. Mathematical models confirm that only compatible strains can stably coexist in unstructured and panmictic host populations when the fitness costs related to infection by different *w*Pip strains are the same [54, 55]. When bidirectional incompatibility inducing *w*Pip strains co-exist in one population the most prevalent strain is expected to eventually invade the population [54, 55] and when unidirectional incompatibility inducing *w*Pip strains co-exist, CI-inducing strain is expected to invade the population once above a frequency threshold [49, 55]. Therefore, a stable co-existence of incompatible strains is predicted to be rare. However, an example of the co-existence of unidirectional incompatibility inducing strains, belonging to a *w*Pip-I group and *w*Pip-IV group, has been shown in Tunisia while mathematical models predicted that *w*Pip-I should have invaded this area in only 4 generations [5, 49]. Atyame et al. [49] hypothesized that low dispersal and extinction-recolonization events could explain this stable co-existence. In one site in Turkey, we observed the co-existence of bidirectional incompatibility inducing strains (*w*Pip-IV and *w*Pip-II). The low prevalence of *w*Pip-IV and its incompatibilities suggest that it should disappear from the population. However, we have evidence that *w*Pip-IV strains were already present at least 13 years ago at Istanbul since it has been sampled in 2003 [35]. This persistence of *w*Pip-IV, at low frequencies, could be explained by higher fitness costs associated with *w*Pip-I and *w*Pip-II infections or by extinction-recolonization events of *w*Pip-IV-infected individuals as it has been suspected in Tunisia [43, 49, 55]. Fitness difference could be, for instance, linked to differences in fecundity [56, 57] or to a possible ability of the different *w*Pip strains to protect their hosts against other microbial infections [58–62]. Further studies on the differences between *w*Pip strains in terms of infection costs and pathogen protection might help to understand stable co-existence of bidirectionally incompatible *w*Pip-IV strains observed in Turkey.

To study the phenotypical diversity of crossing types in Turkey, we crossed Turkish isofemale lines harbouring *w*Pip-I and *w*Pip-II strains with four reference lines defined by Atyame et al. [7]. We inferred both their *mod* and *resc* functions and compared them to the eight *mod* and four *resc* functions already described worldwide. The Tek *w*Pip-I line showed the most common *resc* functions for a *w*Pip-I infected line (i.e. *resc* 2) but a rare *mod* function previously defined in few lines harbouring *w*Pip-I or *w*Pip-II strains (i.e. *mod* “vi”). The Tek *w*Pip-II line showed the same *resc* function as *w*Pip-I (i.e. *resc* 2) but a totally new *mod* function (i.e. *mod* “ix”). Our findings are consistent with theoretical predictions and empirical data suggesting new *mod* functions can more easily evolve and spread in the population than new *resc* functions [7, 63].

Natural CI properties induced by *Wolbachia* can be used to control the vector populations: the mass release of males harbouring incompatible *Wolbachia* into the natural populations can decrease the female reproduction and eradicate the pest/ vector populations (IIT) [29, 30]. Indeed, *w*Pip induced CI has been used against *Cx. quinquefasciatus* (formerly named *Cx. pipiens fatigans*) for the first time in 1967 to control filariasis in Southeast Asia [31]. More recently, natural CI properties caused by *w*Pip infection have been found promising to control *Cx. pallens* (no longer considered as a valid species) in China [32] and *Cx. pipiens* populations in La Réunion Island [33, 34]*.* In the latter study, a *w*Pip-IV strain from Istanbul has been successfully used to sterilise *w*Pip-I females in semi-field conditions. We demonstrated that this Istanbul strain also induces bidirectional incompatibility with mosquitoes harbouring *w*Pip-I or *w*Pip-II in Turkey. This means that most *Cx. pipiens* females in Turkey, except in few sites in the Thrace region, can be sterilised by the release of males infected with Istanbul strain. Although further studies on intrapopulation CI variability, mating choice, hatching rate and population dynamics in semi field populations are needed for *w*Pip-IV Istanbul to be used in future vector control programs in Turkey, a critical region for vector-borne diseases, our results suggest that it could constitute a good candidate.

## Conclusions

We identified *w*Pip diversity in natural *Cx. pipiens* (*s*.*l*.) populations in Turkey. The previously described *w*Pip-IV group was in fact restricted to only two populations while *w*Pip-I and *w*Pip-II group are widely distributed and coexist in many populations all over the country. The *w*Pip-IV strain Istanbul was found bidirectionally incompatible with individuals harbouring *w*Pip-I or *w*Pip-II from Turkey. This highlights the potential of *w*Pip-IV harbouring males as a vector control to sterilise local *Cx. pipiens* populations, particularly where only *w*Pip-I or *w*Pip-II harbouring females were found.

## Abbreviations

C: Compatible
CI: Cytoplasmic incompatibility
IC: Incompatible
*Mod*: Modification ability
*Resc*: Rescue ability
*w*Pip: *Wolbachia pipientis*
